# Cognitive impairment and risk factor prevalence in a population over 60 in Argentina

**DOI:** 10.1590/S1980-57642014DN84000010

**Published:** 2014

**Authors:** Raul L Arizaga, Roxana E Gogorza, Ricardo F Allegri, Patricia D Baumann, María C Morales, Paula Harris, Vicente Pallo, María M Cegarra

**Affiliations:** 1 PRONADIAL (Programa Nacional de Datos, Docencia e Investigación en Alzheimer y otros Trastornos Cognitivos). Departamento de Salud Pública. Facultad de Medicina. Universidad de Buenos Aires, Argentina; 2 Unidad de Investigación en Neurología Cognitiva, Synapse, Buenos Aires, Argentina; 3 Centro de Memoria y Envejecimiento, FLENI, Buenos Aires, Argentina; 4 Hospital Francisco Santojanni, GCBA, Buenos Aires, Argentina

**Keywords:** cognitive impairment, dementia, risk factors, developing countries, Argentina

## Abstract

Epidemiological data on dementia and cognitive impairment are scarce in South America. In Argentina, no dementia/cognitive impairment population-based epidemiological studies are available. The Ceibo Study is a population-based epidemiological study of dementia and cognitive impairment in individuals over 60 to be conducted. The present paper reports the results of the pilot phase (survey of cognitive impairment) conducted in Cañuelas (province of Buenos Aires).

**Methods:**

In a door-to-door survey, trained high school students evaluated 1453 individuals aged 60 years and over in one day using a demographic data and risk factors questionnaire, the Mini-Mental State Examination (MMSE) and the 15-item Geriatric Depression Scale (GDS).

**Results:**

Mean age of the individuals was 70.9 (±7.5) years, 61.4% were women, mean schooling was 5.5 (±3.5) years. Mean MMSE score was 24.5 (±4.7) and mean GDS 3.1 (±2.7). Risk factors of higher prevalence in the population under study were: hypertension (40.6%), smoking (35.1%), alcohol consumption (32.8%), high cholesterol (16.1%), diabetes (12.5%), cranial trauma with loss of consciousness (12.5%), 7 points or more on the GDS (11.7%). Prevalence of cognitive impairment for the whole sample was 23%, and 16.9% in subjects aged 60-69, 23.3% in 70-79 and 42.5% in subjects aged 80 or over . A significant correlation of cognitive impairment with age, functional illiteracy, cranial trauma, high blood pressure, inactivity and depression was found.

**Conclusion:**

In this pilot study, the prevalence of cognitive impairment was comparable with previous international studies.

## Abstract (pt)

Dados epidemiológicos em demência e comprometimento cognitivo são escassos na América do Sul. Na Argentina, não há estudos epidemiológicos de base populacional de comprometimento cognitivo/demência. O Estudo Ceibo é um estudo epidemiológico de base populacional a ser conduzido. O presente artigo reporta os resultados da fase piloto (pesquisa de comprometimento cognitivo) conduzido em Cañuelas (província de Buenos Aires).

**Métodos:**

Numa pesquisa porta-a-porta, estudantes treinados avaliaram 1453 indivíduos de 60 anos ou mais em uma visita, usando dados demográficos e questionários de fatores de risco, o Mini-Exame do Estado Mental (MEEM) e a escala de Depressão Geriátrica (GDS).

**Resultados:**

A idade média foi de 70,9 (±7,5) anos, 61.4% eram mulheres, escolaridade média foi de 5,5 (±3,5) anos. O escore médio do MEEM foi de 24,5 (±4,7) anos e média do GDS de 3,1 (±2,7). Os fatores de risco mais prevalentes foram: hipertensão (40,6%), tabagismo (35,1%), consumo de álcool (32,8%), colesterol elevado (16,1%), diabetes (12,5%), trauma craniano com perda de consciência (12,5%), 7 pontos ou mais no GDS (11,7%). A prevalência de comprometimento cognitive na amostra total foi de 23% e em 16,9% nos sujeitos entre 60-69 anos, 23,3% entre 70-79 e 42,5% naqueles com 80 ou mais. Correlações significativas de comprometimento cognitivo com idade, analfabetismo functional, trauma craniano, hipertensão arterial, inatividade e depressão foram encontradas.

**Conclusão:**

Neste estudo piloto a prevalência de comprometimento cognitivo foi comparável aos estudos prévios internacionais.

## INTRODUCTION

Epidemiological data about dementia and cognitive impairment prevalence are scarce in South America in general and particularly in Argentina.

Ketzoian et al., in Montevideo, Uruguay, found a dementia prevalence of 4.03 per thousand in the general population, with Alzheimer's disease being the most frequent etiology (60%).^1^

In an epidemiologic survey in Catanduva, Brazil, Nitrini et al. found a dementia prevalence of 7.1%. Age, female gender, and low educational level were significantly associated with a higher prevalence of the disease.^2^ The Catanduva cohort follow-up showed a dementia incidence rate of 13.8 per thousand in individuals aged 65 years or older.^3^

Studying an urban population in Concepción, Chile, Quiroga et al. found a dementia prevalence of 5.96% with an incidence rate of 1.78%.^4^

In the population-based Maracaibo Aging Study in Venezuela surveying 3,657 subjects, Maestre et al. demonstrated a dementia prevalence of 8.04%^5^.

The 10/66 Dementia Research Group study found a prevalence of DSM-IV dementia that varied widely in the five Latin American sites (Cuba, Mexico, the Dominican Republic, Venezuela and Peru), ranging from 0.4% (95% CI 0.1-0.9) in rural Peru to 6.3% (5.0-7.7) in Cuba. After standardization for age and sex, the prevalence of DSM-IV dementia in urban Latin American sites was four-fifths of that in Europe (standardised morbidity ratio 80 [95% CI 70-91]). The 10/66 dementia prevalence was higher than that of DSM-IV dementia, and more consistent across sites, varying between 5.7% (95% CI 4.7-6.8) in Venezuela and 11.7% (10.3-13.1) in the Dominican Republic.^6^

As part of the 10/66 Dementia Research Group study, Arizaga et al. conducted the Prevalence Phase of the project in a rural area (Cañuelas) 50 km from Buenos Aires city in a Buenos Aires district, Argentina. Although the study could not be completed, the partial results will be published in the near future.

Pages Larraya et al. in 2004 found cognitive impairment in 23% of subjects over 60 years in Argentina. The sample in the study was not drawn from a selected catchment area and the survey was conducted with volunteers recruited through a "word of mouth" process.^7^

In order to measure the prevalence of both cognitive impairment and dementia to inform health policies related to this problem, an epidemiological population study should be conducted in Argentina.

The characteristics of the Argentine population confer particular importance to the topic. First of all, the real integration of the Spanish colonizers and the natives must be taken into account. From 1813, with slavery abolition, blacks of African descent began to interbreed with the Criollos and Mestizos. From the mid-19th century until the middle of the 20^th^ century, a varied immigration stream took place with marked peaks pre and post First and Second World Wars. The pattern of this immigration shows a wide spectrum. Between 1857 and 1950 Argentina received 111,087 Turks, Arabs and Syrians, 137,847 French, 167,694 Poles, 1,274,000 Spaniards and 1,733,726 Italians. These new contingents were widely accepted and quickly mixed with the existing population. The result of this prolonged and sustained process of integration is the current population of Argentina: broad in its genotypic composition and narrow in phenotypic expression.^8^

With regard to lifestyle and food habits, the Argentine population has certain peculiarities. Argentines are one of the biggest beef consumers in the world. Argentina's per capita wine consumption is the highest in America, being comparable to the high consumption European countries.

A dementia and cognitive impairment population study in a community of mixed origin with a high component of Caucasian features but with particular eating habits will, through the prevalence, incidence and risk factor data gathered, yield two significant results. One is to determine the actual local situation with respect to cognitive impairment and dementia with all preventive, diagnostic, therapeutic and economic implications for the design and planning of a national social and health action. Another is, through comparative analysis of risk factors, to contribute to the international knowledge on the effect of environmental factors linked to lifestyle on the development, natural history and evolution of cognitive impairment and dementia.

The Ceibo study is a population-based, epidemiological study of dementia and cognitive impairment in over 60-year-olds. It is designed to be conducted in three phases: first phase (survey of cognitive impairment in the population older than 60 years), second phase (clinical and neuropsychological diagnosis,) and third phase (longitudinal study).

The aim of this paper was to show the results of a pilot of first the phase of the study (survey of cognitive impairment in individuals older than 60 years).

## METHODS

This study was conducted in Cañuelas, Buenos Aires province. Cañuelas, capital of the county bearing the same name, is a city located 50 km from Buenos Aires. The last national census of the population (2001) living in Cañuelas recorded 42,475 inhabitants (21,235 men and 21,240 women).

The economic activity of the town is related to agricultural production and livestock rearing.

One hundred and twenty high school students were trained, twice a week, for three months. Upon completion of the training, 88 pupils were able to administer the survey questionnaire to individuals over 60 in the catchment area. The population was made aware about the survey through local media ads, flyers and pre-survey visits. On the day of the survey, the students bearing identity badges and covered by insurance, performed the survey from 8 am to 4 pm, house by house in the catchment area. In this door-to door survey, 4,768 households were visited where 3,122 individuals over 60 were contacted, 1,526 of whom agreed to complete the survey. After exclusion of 73 forms (incomplete or illegible), the final sample comprised 1,453 interviews.

**Instruments.** [a] a demographic data and risk factors questionnaire was completed by participants; [b] the Mini-Mental State Examination,^9^ version adapted for Buenos Aires,^10^ was used to select those subjects with probable cognitive impairment. Suitable cut-off values were selected for the demographic characteristics of the population under study (Table 1). A mean age of 70.8 (+7.5) years and mean schooling of 5.53 (+ 3.5) years were observed. Based on the paper published by our group in 2001,^11^ the MMSE cut-off for the sample was established at 22 points (the most suitable for age and schooling averages); [c] the 15-item questionnaire of the Geriatric Depression Scale (GDS).^12^ Scale scores of 6 or less were considered as no depression. Scores 7 or greater were considered as an indicator of depression.

**Table 1:** Sample data.

Total participants	1453
Age	70.9 (±7.5)
Sex (male / female)	560 / 893
Years of schooling	5.5 (±3.5)
Handedness	
• Right	87.4%
• Left	6.4%
• Ambidextrous	6.2%
Activity	
• Retired	85.3%
• Active	14.7%
Retirees’ previous occupation	
• Professional	1.6%
• Entrepreneur / Businessman	7.2%
• Employee	21.2%
• Specialized worker	2.7%
• Unspecialized worker	10.9%
• Teacher	2.4%
• Homemaker	44.5%
• Other	9.5%
Geriatric Depression Scale (0-15) average score	3.1 (±2.7)
MMSE (0-30) average score	24.5 (±4.7)

Age, years of schooling, GDS score and MMSE score are mean values (standard deviation in brackets). Total participants and distribution by sex are absolute values. The remainder are expressed in percentages.

**Statistical analysis.** SPSS for Windows software, Standard Version (1999) was employed. The descriptive statistics of the population under study was expressed as mean and standard deviations. The frequency of occurrence of each risk factor studied in this sample was calculated.

Bivariate analyses were performed according to the type of variables. For nonparametric data, the Kruskal Wallis and Chi-square tests were used, whereas for parametric data the p-Student (t-test) test, or the test of analysis of variance (ANOVA) was employed. For correlations between variables, Pearson's parametric test and Spearman's nonparametric test were used. For the multivariate analysis, a regression model was constructed. Based on the statistical analysis described previously, the sample demographic characteristics are given in Table 1.

## RESULTS

This sample comprised 893 women (61.4%) and 560 men.

Adopting 22 points as the cut-off selected for the Mini-Mental State Examination^10,11^ according to the age and schooling of the study sample, based on MMSE average score a total of 352 (23.2%) subjects with cognitive impairment were detected (Table 3). Stratification by age showed increasing deterioration prevalence with increasing age: 16.9% in the 60-69 years band; 23.3% in 70-79 years and 42.5% in the 80 years and over age group. Analysis of the sample according to sex revealed no difference in the prevalence of cognitive impairment by gender.

**Table 2:** Distribution of population according to presence or otherwise of cognitive impairment (adopting MMSE cut-off score of 22).

	No impairment	With impairment	Significance
Number of individuals	1101	352	
Age	69.7 (6.7)	73.8 (8.3)	p<0.01^#^
Sex (% male)	35.5%	33.5%	ns*
Years of schooling	6.1 (3.3)	4.2 (3.1)	p<0.01^#^
Depression (GDS> 7)	2.8 (2.5)	3.6 (3.0)	p<0.01^#^
MMSE score	26.7 (2.1)	18.0 (4.2)	p<0.01^#^
Active / Retirees	15.2%	11.0%	ns*
Hypertension	38.7%	44.0%	*p<0.01
Dyslipidemia	16.1%	15.6%	ns*
Diabetes	11.1%	13.3%	ns*
Head trauma	10.8%	16.4%	p<0.01*
Smoking	34.4%	35.7%	ns*
Alcohol consumption	33.9%	30.6%	ns*

Age, years of schooling , GDS and MMSE scores are expressed as mean and standard deviations. Remaining values are in prevalence percentage.

# Statistical processing was performed with the t-test for parametric variables;

* Kruskall Wallis and Chi-square tests for non-parametric variables

**Table 3:** Correlation between cognitive impairment (presence or absence) and demographic variables and risk factors in sample.

	Correlation with cognitive impairment	Significance
Age	–0.228	P<0.01
Sex	–0.046	Ns
Years of schooling	0.295	P<0.01
Depression	–0.157	P<0.01
Hypertension	0.075	P<0.01
Use of tranquilizers	0.001	Ns
Dyslipidemia	–0.006	Ns
Diabetes	–0.004	Ns
Head trauma	0.109	P<0.01
Smoking	0.023	Ns
Alcohol consumption	0.010	Ns

Based on the cut-off value, the sample was divided into two populations: one group with no cognitive impairment and one group with cognitive impairment. Comparative analysis of these populations showed significant differences in age (subjects with impairment were older), education (impaired subjects had less schooling) and depression scale (impaired individuals had higher prevalence of depression). Of the risk factors analyzed, head trauma and high blood pressure were significantly more frequent in patients with cognitive impairment (Table 2).

Different risk factors for cognitive impairment / dementia were reported by the interviewees. The most prevalent factors in the sample were hypertension (40.6%), smoking (35.1%) and alcohol consumption (32.8%). Other reported conditions were dyslipidemia (16.1%), diabetes (12.5%) and head trauma (12.5%). Depression was consider present in individuals with 7 or more points on the Geriatric Depression Scale of Yesavage (11.7%).

In order to better elucidate the relationship of cognitive decline with age, schooling and the presence of indicators of depression, a p-Pearson's correlation test for variables was used on the results of the Mini-Mental State Exam (cognition) and age, years of schooling and depression scale score. For the risk factors, Spearman's non-parametric correlation test was utilized. A significant direct correlation with schooling was found: greater schooling in years was associated with better results on the MMSE (ergo less cognitive impairment). Results were also highly significant on the analysis of age effects: older persons obtained poorer results on the MMSE (further deterioration). The same applied to the presence of depression: higher scores on Yesavage's depression scale (stronger indicator of depression) coincided with lower scores on the MMSE (greater cognitive impairment). The most significantly correlated risk factors were hypertension and head trauma. (Table 3).

To evaluate the importance of possible risk factors for cognitive impairment using another statistical method, multivariate analysis with a regression model in which the dependent variable was the presence or absence of cognitive impairment, was conducted. The absence of cognitive impairment was categorized as 0 (zero) and the presence of impairment as 1 (one), (based on the MMSE cut-off of 22). It is again evident that the possibility of cognitive impairment increases with age and decreases with higher levels of schooling. Head trauma and lack of activity in the elderly were statistically significant risk factors for cognitive impairment (Table 4).

**Table 4:** Regression model using cognitive impairment (absence vs presence) as dependent variable.

Primary independent variable	Regression coefficient B	T	P
Age	–0.227	–5.894	< 0.01
Sex	–0.030	–0.786	Ns
Years of schooling	0.199	5.628	<0.01
Hypertension	0.030	0.857	Ns
Active / Retired	0.077	2.018	< 0.05
Dyslipidemia	–0.002	0.058	Ns
Diabetes	0.050	1.425	Ns
Head trauma	0.089	2.546	<0.05
Smoking	0.066	1.868	Ns
Alcohol consumption	–0.002	0.050	Ns
Depression	–0.048	1.378	Ns

Results showed that 11.7% of the total population studied presented depression (according to the detection of depression by Yesavage's questionnaire). There was evidence of a significant correlation between cognitive impairment and depression (r= -0.157, p<0.01). There was also correlation of depression with age (r= -0.026), sex (r=0.091) and schooling level (r=0.053).

For this reason it was decided to observe the influence of the different factors in a population without indicators of depression. Of the total population under study (1,453 subjects) 171 subjects whose average score on Yesavage's questionnaire detecting depression was less than or equal to 6 to were excluded. Thus, a total of 1,277 individuals without GDS depression indicators were analyzed. Dividing the sample according to the presence or absence of cognitive impairment, 288 had an MMSE score lower than 22. A regression model was devised using presence and absence of deterioration as the dependent variable. The results revealed that in the absence of depression variables predicting cognitive impairment were age, low schooling, passive life and head trauma (Table 5).

**Table 5:** Regression model: Cognitive impairment (absence vs presence) as dependent variable excluding individuals with depression (GDS > 7).

Primary independent variables	Regression coefficient B	T	Significance
Age	–0.195	–4.683	<0.01
Sex	–0.055	–1.224	Ns
Years of schooling	0.197	5.248	<0.01
Active / Retired	0.080	1.960	<0.05
Hypertension	0.030	0.784	Ns

## DISCUSSION ON RISK FACTORS

**Age.** The study results showed a clear correlation between age and increasing prevalence of cognitive impairment. This finding corroborates the majority of international studies.

**Educational level.** The correlation demonstrated in this study (lower schooling level, higher prevalence of cognitive impairment) has been previously reported in the international literature.

Using the MMSE, Kramer^13^ found severe cognitive impairment in 12.5% of a group with lower educational level against 1.5% of another group with higher level. For the study of Weissman^14^ using the same instrument, the equivalent values were 6.1% and 0.2%, respectively.

The study by Yu et al.^15^ in China found 11.4% severe cognitive impairment in illiterates against only 0.4% in individuals who had completed high school. O'Connor's study^16^ in Great Britain indicated cognitive impairment in 12% of individuals who had dropped out of school after the age of 15 compared with 23% of those who had left before that age. Other studies indicate that there is no probable correlation between MMSE and educational level.^17-21^

The interaction of various factors such as increased frequency of disease, poor nutrition (fetal and infant) with consequent poor brain development (less reserve against neuronal loss of aging), health habits, poor diet and less mental stimulation is postulated as being responsible for this higher prevalence of cognitive impairment in individuals with low educational level.^22^ In the Nun Study (clinical, genetic, pathological study) being conducted in 600 Catholic nuns, published findings showed that cognitive impairment as measured with the MMSE, decreased more markedly in the group with less education.^23^ Severe cognitive impairment was twice as high in the Sisters with a low level of education compared to individuals with a higher educational level.^24^

**Hypertension.** The study showed a higher prevalence of hypertension in individuals with cognitive impairment compared with cognitively normal subjects.

Hypertension is the major risk factor for stroke and cognitive impairment of vascular origin.^25-30^ In the present study, statistically significant differences were detected in the prevalence of hypertension in the sample when stratified by sex.

**Wine consumption.** High consumption of alcohol contributed to overall all-cause and cardiovascular mortality. Moderate consumption appears to decrease general-cause mortality, mortality from coronary heart disease and risk of stroke.^31^ In relation to Alzheimer's disease, it is very difficult to assess the consumption of alcohol as a risk factor because the diagnosis of the disease involves the exclusion of alcoholism.^32^

In Bordeaux, France, a prospective study^33^ showed a lower incidence of dementia (OR=0.19) and AD (OR=0.28) in subjects categorized as moderate wine drinkers (250 to 500 ml/day). In the present analysis, wine consumption was found to be neither a risk factor nor protective factor for incidence of cognitive impairment. It should be taken into account that in this study no exhaustive and detailed investigation about the topics in the survey form was carried out. Also the possible tendency of respondents to under-report the consumption of alcoholic beverages in general should be considered. Since some international publications, several of which are outlined below, report conflicting findings on alcohol as a risk factor for dementia and wine as a protective factor for cognitive impairment, further investigation of this issue is necessary in future phases of the study.

**Head trauma.** In the present study, there was a clear correlation between the occurrence of head trauma with loss of consciousness and the prevalence of cognitive impairment. Head trauma has been implicated as a risk factor for Alzheimer's disease.^34,35^ In a case-control study^36^, 25.3% of dementia patients had a history of head trauma with loss of consciousness. In the EURODEM analysis, seven studies focused on head trauma as risk factor in a pool of 1,059 cases and 1,059 controls. A significant effect was found, with an odds ratio (OR) of 1.82.^37,38^ Therefore, these data have now established head trauma as a possible risk factor for Alzheimer's disease. Subsequent studies suggest that TBI is a risk factor only in individuals with genetic predisposition related to APOE 4 alleles.^39^ A history of head trauma, coupled with the presence of the E4 allele, multiplied the risk of developing Alzheimer's disease tenfold, while in the absence of the E4 allele risk was not increased.

**Diabetes.** In this sample, a very slight correlation between presence of diabetes and existence of cognitive impairment was evident. Although previous studies have shown no statistically significant association between AD and diabetes, a prospective study^40^ showed that individuals with type 2 diabetes had almost double the risk of dementia (RR=1.9) and AD (RR=1.9). Insulin-treated patients had a higher relative risk (RR=4.3). Alterations in insulin production and in the receptor of insulin (RI) activity in the brain causes deficits in learning and memory.

**Smoking.** This analysis failed to demonstrate a relationship between cognitive impairment and smoking (current or past). Analyzing smoking as a risk factor for AD, one study found a lower incidence of smoking in cases than in controls.^41^ The meta-analysis performed by Mortimer^42^ with data from seven case-control studies, indicated that a tobacco-AD association was improbable. Other case-control and longitudinal studies^43,44^ as well as reviews^45,46^ suggest a possible protective effect of cigarettes for AD. The Rotterdam^40^ study found that smoking doubles the risk for dementia and AD. In London, the results of a longitudinal study^47^ indicated that smokers had a RR=37 for cognitive impairment compared with non-smokers.

To conclude, the present survey showed a cognitive impairment prevalence of 23% in a population of 1,453 individuals aged 60 and over. In the present study, the cause of cognitive impairment was not determined (emotional and/or psychosocial factors, organic vascular or neurodegenerative origin). Older age and low schooling correlated with higher prevalence of cognitive impairment. The most prevalent medical conditions in subjects older than 60 years of age with cognitive impairment were hypertension and head trauma. Detection of depression indicators correlated with higher prevalence of cognitive impairment.
